# Telehealth requires improved evidence to achieve its full potential in palliative care

**DOI:** 10.1177/02692163231182461

**Published:** 2023-06-30

**Authors:** Amara Callistus Nwosu

**Affiliations:** 1 Lancaster Medical School, Lancaster University, Lancaster, UK; 2 Marie Curie Hospice Liverpool, Liverpool, UK; 3 Liverpool University Hospitals NHS Foundation Trust, Liverpool, UK

## Telehealth has unrealised potential in palliative care

Palliative care telehealth (describing the use of technology for healthcare access) and telemedicine (describing use of technology for care delivery) is increasingly being used in the delivery of palliative care.^1,2^ Palliative care telehealth is increasing, due to advancements in technological hardware, software and infrastructure (e.g., upgrades to battery technology, sensors, portable computing and wireless internet coverage);^3,4^ however, evidence of its efficacy and effectiveness is limited.^5^ The COVID-19 pandemic caused many palliative care services to rapidly implement telehealth services to reduce face-to-face human contact to limit virus transmission.^1^ Examples of palliative care telehealth services implemented during COVID-19 include clinical monitoring, patient assessment, psychological support, spiritual care and day therapy services.^6–14^ The World Health Organisation has recently announced that COVID-19 is no longer a global health emergency,^15^ however, its impact in increasing palliative care telehealth will likely remain, partly due to increasing global need for palliative care, meaning technological innovation is needed to provide care.^16^

## Potential opportunities for palliative care telehealth

Palliative care telehealth offers potential to improve access and choice for patients.^5^ For example, telehealth could facilitate more efficient time management for the multidisciplinary team. Improved management of resources could streamline (and reduce) the number of face-to-face clinical appointments for patients. Telehealth could improve access to palliative care services for people in areas without access specialist palliative care services (e.g., remote areas communities), and support the identification of people requiring specialist evaluation and treatment (e.g., emergency oncological treatment for presumed metastatic cord compression). From a service level, telehealth could help organisations to support staff coordination, education and pastoral care. Finally, novel data technologies provide new possibilities to innovate care through its incorporation with telehealth,^17^ for example artificial intelligence (e.g., natural language processing of free text notes), audio (e.g., speech pattern analysis) and visual (e.g., facial gestures analysis), which could potentially provide the clinician with data that adds value to the consultation.

## Potential challenges for palliative care telehealth

Many palliative care telehealth interventions lack detailed evaluation in real-world settings, meaning their usefulness (and transferability) is uncertain.^5,18^ Governments commonly cite cost reduction, improved self-management of illness and homecare delivery as reasons to adopt telehealth systems.^19^ However, this premise is potentially troublesome when considering palliative care needs, as telehealth may lead to *increased* intensity of face-to-face support as physical function worsens. In this situation, telehealth may (appropriately) identify those who require more in-person support, following which telehealth may no longer be possible as the individual may no longer be able to use telehealth systems due to their declining health. Without long-term economic analysis, there is risk that some telehealth programmes (implemented rapidly during COVID-19) were not costed appropriately, meaning details of long-term running costs (e.g., infrastructure and personnel) are unknown. Telehealth systems may increase the risk of bias towards some users if (as noted in algorithmic studies) technology is developed with data unrepresentative of certain communities (e.g., cultural and language minorities).^20^ A failure to adequately design telehealth systems for the changing requirements of people with palliative care needs may cause them to be deemed expensive, ineffective and inefficient.

## Future actions need in telehealth palliative care research

Evidence to identify how telehealth services should be used (including appropriate models of care and economic considerations) are needed for this technology to achieve its full potential in palliative care. Interdisciplinary research partnerships (for example, including designers, economists and ethicists) should be conducted, to adequately research the areas needed to determine how telehealth can be used, safely and effectively, to improve palliative care for patients and caregivers.
